# Leukocytosis and neutrophilia predict outcome in locally advanced esophageal cancer treated with definitive chemoradiation

**DOI:** 10.18632/oncotarget.14584

**Published:** 2017-01-10

**Authors:** Antoine Schernberg, Laurence Moureau-Zabotto, Eleonor Rivin Del Campo, Alexandre Escande, Michel Ducreux, France Nguyen, Diane Goere, Cyrus Chargari, Eric Deutsch

**Affiliations:** ^1^ Radiotherapy Department, Gustave Roussy Cancer Campus, Villejuif, France; ^2^ Radiation Oncology Department, Institut Paoli-Calmettes, Marseille, France; ^3^ Université Paris Sud, Université Paris Saclay, Faculté de médecine du Kremlin-Bicetre, Le Kremlin-Bicetre, France; ^4^ Department of Medical Oncology, Gustave Roussy, Université Paris-Saclay, Villejuif, France; ^5^ Department of Surgery, Gustave Roussy, Université Paris-Saclay, Villejuif, France; ^6^ INSERM1030, Gustave Roussy Cancer Campus, Villejuif France; ^7^ French Military Health Services Academy, Ecole du Val-de-Grâce, Paris, France; ^8^ Institut de Recherche Biomédicale des Armées, Bretigny-sur-Orge, France

**Keywords:** esophageal cancer, concurrent chemoradiation, prognostic factor, biomarker, leukocytosis

## Abstract

**Purpose:**

To investigate the prognostic value of leukocyte and neutrophil count as biomarkers in patients with locally advanced esophageal squamous cell carcinoma (SCC) undergoing exclusive chemoradiation.

**Results:**

A total of 126 patients were identified. Respectively, 33% and 35% displayed baseline leukocytosis and neutrophilia. Estimated 3-year OS and PFS from chemoradiation completion were 31% and 25%, respectively. In univariate analysis, both leukocytosis and neutrophilia were associated with worse OS, PFS, and LRC (*p* < 0.01). In multivariate analysis, leukocytosis remained an independent risk factor associated with poorer OS, PFS and LRC (*p* < 0.05), independently from tumor stage and length, with higher prognostic value for OS compared with patients’ performance status (PS).

**Materials and Methods:**

Bi-institutional clinical records from consecutive non-operable patients treated between 2003 and 2015 with definitive chemoradiation for locally advanced esophageal carcinoma were reviewed. Leukocytosis and neutrophilia were defined as a leukocyte or neutrophil count over 10 G/L and 7 G/L, respectively. These parameters were studied for their potential correlation with overall survival (OS), progression free survival (PFS), locoregional control (LRC) and distant metastases control (DMC).

**Conclusions:**

Leukocytosis and neutrophilia were independent prognostic factors of poor OS, PFS, and LRC in this bi-institutional series of locally advanced esophageal SCC treated with definitive chemoradiation. Although prospective confirmation is warranted, it is suggested that the leukocyte and neutrophil count parameters might be clinically relevant biomarkers to be considered for further clinical investigations.

## INTRODUCTION

Esophageal cancer is the eighth most common cancer worldwide, with an estimated 456,000 new cases in 2012, and the sixth most common cause of death from cancer with an estimated 400,000 deaths in 2012, with both incidence and mortality increasing in the US, whereas the incidence and mortality of other cancers have decreased [1]. There are two common histologies of esophageal cancer: squamous cell carcinoma (SCC) and adenocarcinoma (ADK). Both histological subtypes are usually included in clinical trials, despite the extensive evidence supporting significant differences in terms of pathogenesis, epidemiology, tumor biology, and prognosis [2]. To date, the prognosis of patients with esophageal carcinoma remains particularly poor with a median progression free survival (PFS) of 9.7 months in the recent phase 2/3-study PRODIGE5/ACCORD17, despite optimization of the treatment planning process and integration of systemic agents to improve treatment efficacy [3]. Identification of new biomarkers would be therefore crucial, allowing to better identify patients who might benefit from dose escalation or intensification of systemic therapies.

Biological pathways involved in inflammation and immunity play a critical role in tumorigenesis, and conversely cancer promotes inflammation [4]. Tumor-related leukocytosis and particularly neutrophilia are a paraneoplastic syndrome reported in various malignant advanced tumor types [5]. The expression of cancer cell-derived granulocyte-colony stimulating factor (G-CSF) may enhance neutrophilia through an aberrant paracrine activity and intratumoral chemotaxis of myeloid-derived suppressor cells [6].

Hematological inflammatory biomarkers are inexpensive and easy to perform, already used in daily oncologic practice as part of routine pretreatment investigations. The systemic inflammatory response, which is usually measured by peripheral blood-based parameters such as C-reactive protein, neutrophil, lymphocyte or platelet count, has been shown to be a predictive factor in various cancers, including esophageal [7]. Neutrophil lymphocyte ratio (NLR) seems associated with tumor progression and poorer survival in patients with esophageal cancer treated with surgery, but this finding is controversial in patients undergoing definitive chemoradiation [8]. However, it is now well documented that ionizing radiation activates several transcription factors modulating the expression of mediators in tumor cells and cells of the microenvironment, which could explain this inconsistency [9].

Based on the evidence that prognostic stratification of esophageal carcinoma patients needs to be improved, this study investigated the prognostic value of leukocyte and neutrophil count in patients with esophageal SCC undergoing exclusive chemoradiation in a bi-institutional retrospective series.

## RESULTS

### Patients and tumors

A total of 126 patients with locally advanced esophageal SCC unsuitable for surgery were included for analysis. Median age was 62 years (range: 22–86 years). Median tumor length was 5 cm (range: 1–14 cm). Tumor length was over 7 cm in 32 patients (25%). One hundred and twenty-five patients (99%) had locally-advanced disease defined as T3 tumors (transmural extension), T4 tumors (invasion of adjacent structures), or N1-2 (lymph node-positive) disease. Patient's performance status (PS) were PS 0 or 1 in 109 patients (87%) according to the World Health Organization (WHO) classification.

On initial blood count, before the first week of EBRT, median leukocyte and neutrophil counts were 8.7 G/L (2.9–23.3 G/L) and 5.8 G/L (1.1–21.2 G/L), respectively. Leukocytosis and neutrophilia were found in 41 patients (33 %) and 44 patients (35 %), respectively. Characteristics of patients, tumors and blood cell count results are shown in Table 1.

**Table 1:** Patient and treatment characteristics

overall population *n* (% or median[range])		PNN < 7 G/L *n* (% or median[range])	PNN ≥ 7 G/L *n* (% or median[range])		*p*
**Total**		126	81	44	
**Hospital**	GR	71 (56%)	38 (47%)	32 (73%)	*0.010*
	PCI	55 (43%)	43 (53%)	12 (27%)	
**Age**		62 [22, 86]	63 [22, 86]	61 [46, 82]	*0.328*
**PS**	0	36 (29%)	23 (28%)	12 (27%)	*0.519*
	1	73 (58%)	49 (61%)	24 (55%)	
	2	16 (13%)	8 (10%)	8 (18%)	
	3	1 ( 1%)	1 (1%)	0 (0%)	
**Gender**	Female	31 (25%)	22 (27%)	8 (18%)	*0.366*
	Male	95 (75%)	59 (73%)	36 (82%)	
**Location (1/3)**	Inferior	30 (24%)	21 (26%)	9 (21%)	*0.128*
	Middle	56 (44%)	31 (38%)	25 (57%)	
	Superior	40 (32%)	29 (36%)	10 (23%)	
**Tumor length**		5 [1, 14]	5 [1, 10]	5.50 [2, 14]	*0.005*
**Tumor stage**	T1	5 (4%)	4 (5%)	1 (2%)	*0.030*
	T2	10 (8%)	10 (12%)	0 (0%)	
	T3	97 (77%)	61 (75%)	35 (80%)	
	T4	14 (11%)	6 (7%)	8 (18%)	
**Nodal involvement**	Negative	10 (8%)	8 (10%)	2 (5%)	*0.407*
	Positive	114 (90%)	71 (86%)	42 (96%)	
	unknown	2 (2%)	2 (1%)	0 (0%)	
**Hemoglobin (g/dL)**		13.40 [8.0, 17.1]	13.40 [8.4, 17.1]	13.3 [8, 16.^4^]	*0.660*
**Platelets (G/L)**		271 [50, 1283]	247 [149, 398]	316 [50, 1283]	*0.007*
**Leukocytes (G/L)**		8.7 [2.9, 23.3]	7.6 [2.9, 14.7]	11.8 [8.8, 23.3]	*< 0.001*
**Neutrophils (G/L)**		5.8 [1.1, 21.2]	4.7 [1.1, 6.9]	9.1 [7, 21.2]	*< 0.001*
**Lymphocytes (G/L)**		1.70 [0.20, 5.70]	1.7 [0.5, 5.7]	1.5 [0.2, 4.6]	*0.217*
**Monocytes (G/L)**		0.66 [0.19, 3.10]	0.6 [0.2, 1.5]	0.8 [0.4, 3.1]	*< 0.001*
**Leukocytosis**	Yes	41 (33%)	3 (4%)	38 (86%)	*< 0.001*
	No	84 (67%)	78 (97%)	6 (14%)	
	unknown	1 (1%)	0 (0%)	0 (0%)	
**Neutrophilia**	Yes	44 (35%)	0 (0%)	44 (100%)	*-*
	No	81 (64%)	81 (100%)	0 (0%)	
	unknown	1 (1%)	0 (0%)	0 (0%)	
**NLR**		3.58 [0.70, 94.90]	2.6 [0.7, 8.7]	6 [2.2, 94.9]	*< 0.001*
**Concurrent CT**	0	4 (3%)	3 (4%)	1 (2%)	*1.000*
	1	122 (97%)	78 (96%)	43 ( 98%)	
**CT regimen**	FOLFOX	65 (52%)	42 (52%)	22 (50%)	*0.774*
	5FU + CDDP	37 (29%)	22 (27%)	14 (32%)	
	Platinum	18 (14%)	13 (16%)	6 (14%)	
	Other	3 (2%)	2 (3%)	1 (2%)	
	No CT	3 (2%)	2 (3%)	1 (2%)	
**Treatment duration**		40 [17, 98]	42 [25, 98]	37 [17, 58]	*0.010*
**IMRT**	No	93 (74%)	57 (70%)	35 (79%)	*0.369*
	Yes	33 (26%)	24 (30%)	9 (21%)	
**PTV dose**		50.4 [22, 66]	50.4 [28.8, 66]	50 [22, 60]	*0.018*
**2nd cancer**		6 (5%)	6 (7%)	0 (0%)	*0.158*

PS: Performance Score; 5FU: 5-fluorouracil; CDDP: Cisplatin; CT: chemotherapy; IMRT: Intensity-Modulated Radiotherapy; FOLFOX: combination regimen of 5-fluorouracil and oxaliplatin; NLR: Neutrophil Lymphocyte Ratio; PTV: Planning Target Volume.

### Treatment

All patients received thoracic irradiation, delivered with 3D-conformal technique for 93 patients (74%) and IMRT for 33 patients (26%). There was no significant impact of the technique on OS (*p* = 0.955) or PFS (*p* = 0.912). Median total dose to the PTV was 50.4 Gy (22–66 Gy) with a median treatment duration of 40 days (17–98 days).

Most patients (98 %) received concurrent chemotherapy, based on FOLFOX in 65 patients (52%), and 5FU-cisplatin in 37 patients (29%). Eighteen patients (14%) unfit for these regimens received carboplatin or cisplatin monotherapy, while 3 patients (2%) received other regimens (cetuximab in 1 patient, paclitaxel in another and docetaxel in another). Mean hemoglobin (*p* = 0.335), platelet (*p* = 0.182), leukocyte (*p* = 0.976), neutrophil (*p* = 0.925), lymphocyte (*p* = 0.836) and monocyte (*p* = 0.931) counts were not significantly different in patients that received FOLFOX, 5FU-cisplatin or other regimens. There was no difference in concurrent chemotherapy regimens between patients with baseline leukocytosis or neutrophilia vs. others (*p* = 0.776 and *p* = 0.774 respectively). Treatment characteristics are reported in Table 1.

### Outcome

Median follow-up of the whole cohort was 12 months (range: 2 to 127 months), relapses were reported in 88 patients (70%). Locoregional relapses occurred in 54 patients (43%), distant metastases in 32 patients (25%).

At last follow-up, 81 patients (64%) had died. Estimated 3-year OS was 31% (95%CI: 23–41%). Estimated 3-year PFS was 25% (95%CI: 18–35%).

### Blood count disorders

Patients who had died from tumor evolution at the time of analysis had higher initial leukocyte count (*p* < 0.001), neutrophil count (*p* < 0.001) and monocyte count (*p* = 0.002). No statistically significant difference was seen regarding hemoglobin levels, platelet count, or lymphocyte count. Patients with baseline leukocytosis or neutrophilia displayed a worse initial disease, with higher T-stage (*p* = 0.030) and tumor length (*p* = 0.005). Patients with a worse PS were older (*p* = 0.022), and had a longer tumor length (*p* = 0.027). All correlations between baseline and treatment characteristics are displayed in Supplementary Figure S1.

### Prognostic value of leucocyte disorders

**Figure 1:**
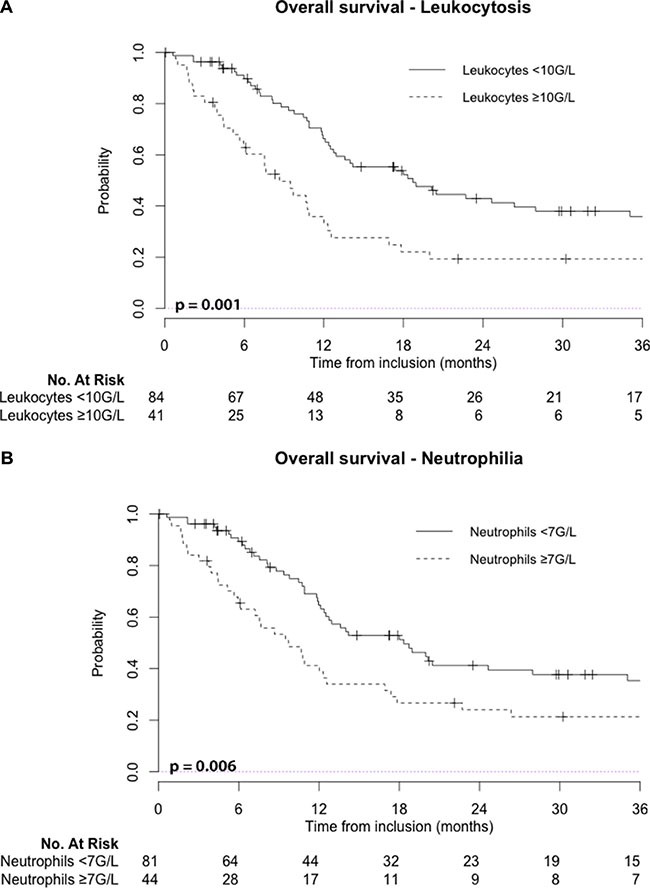
Leukocytosis: leukocyte count ≥ 10 G/L; Neutrophilia: neutrophil count ≥ 7 G/L; No: number

In univariate analysis significant factors associated with worse OS were leukocytosis (*p* = 0.001), neutrophilia (*p* = 0.006), thrombocytosis (*p* = 0.034), monocytosis (*p* = 0.017), higher PS (*p* < 0.001), T3-4 vs. T1-2 (*p* = 0.014), and tumor length > 7 cm (*p* < 0.009). Neither anemia, lymphopenia, age, gender, tumor localization, nodal involvement, chemotherapy regimen or radiotherapy duration significantly predicted OS (*p* > 0.10). On the other hand, the Neutrophil to Lymphocyte ratio > 3.5 (NLR) also correlated with worse OS (*p* = 0.010). At 2-years, estimated OS was 43% (95%CI: 33–56%) for patients without initial leukocytosis vs. 19% (95%CI: 10–37%) for patients with initial leukocytosis, and similarly estimated 2-year OS was 41% (95%CI: 31 - 55%) vs. 24% (CI95%: 14 - 41%) regarding neutrophilia (Figure 1).

**Figure 2:**
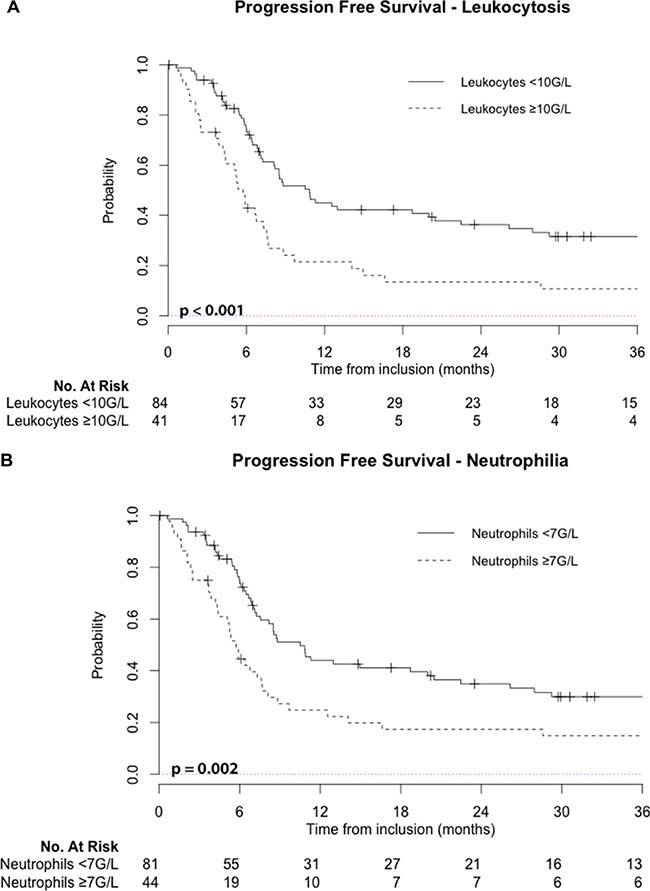
Leukocytosis: leukocyte count ≥ 10 G/L; Neutrophilia: neutrophil count ≥ 7 G/L; No: number

Using univariate analysis, pre-treatment leukocytosis and neutrophilia were also significant prognostic factors for PFS (*p* < 0.001 and *p* = 0.002, respectively). At 2-year follow-up, estimated PFS was 36% (95%CI: 27–49 %) for patients without initial leukocytosis vs. 13% (95%CI: 6–30 %) for patients with initial leukocytosis (Figure 2).

Leukocytosis and neutrophilia were also associated with worse LRC (*p* < 0.001), and leukocytosis also predicted worse DMC (*p* = 0.049). Supplementary Figures S2 and S3 show respectively locoregional control and distant metastasis control regarding baseline leukocytosis or neutrophilia.

Using multivariate analysis, leukocytosis and neutrophilia were independent factors of poorer OS with a hazard ratio (HR) of 2.01 and 1.71, respectively. They were also independently associated with worse PFS (HR 2.35; HR 1.97, respectively) and LRC (HR 1.99; HR 1.68, respectively). Tumor length independently predicted poorer PFS, LRC and DMC, while higher PS was the only clinical parameter independently associated with both poorer OS, PFS, LRC and DMC (Table 2).

**Table 2:** Results of univariate and multivariate Cox analyses

Variable	Overall Survival								Progression Free Survival					
	Univariate (HR[CI])		*p*		Multivariate (HR[CI])		*p*		Univariate (HR[CI])	*p*		Multivariate (HR[CI])		*p*
**Leukocytosis**	2.13 (1.36–3.33)		0.001		2.01 (1.25–3.25)		0.004		2.18 (1.42–3.34)	< 0.001		2.35 (1.46–3.78)		**< 0.001**
**Neutrophiliaa**	1.85 (1.19–2.87)		0.006		1.71 (1.03–2.82)		0.037		1.97 (1.29–3.02)	0.002		1.97 (1.18–2.27)		**0.001**
**Monocytosisa**	1.99 (1.18−3.36)		0.017		−		0.089		2.71 (1.62–4.56)	< 0.001		1.90 (1.11 − 3.25)		**0.020**
**Anemia**	−		0.710		−		−		−	0.707		−		**−**
**Lymphopenia**	−		0.734		−		−		−	0.960		−		**−**
**Age**	−		0.650		−		−		−	0.844		−		**−**
**PS**	1.82 (1.33 − 2.47)		< 0.001		1.82 (1.31 − 2.51)		0.001		2.04 (1.42 − 2.91)	< 0.001		2.11 (1.50 − 2.98)		**< 0.001**
**T length > 7cm**	1.89 (1.17 − 3.05)		0.009		−		0.101		2.53 (1.55 − 4.15)	< 0.001		1.87 (1.14 − 3.07)		**0.013**
**T stage**	1.56 (1.08 − 3.05)		0.014		1.75 (1.08 − 2.82)		0.022		1.55 (1.04 − 2.29)	0.029		−		**0.051**
**N+ status**	−		0.397		−		−		−	0.069		−		**0.297**
**IMRT**	−		0.955		−		−		−	0.710		−		**−**
**Tr. duration**	−		0.167		−		0.974		−	0.163		−		**0.856**
**RT dose**	−		0.089		−		−		−	0.284		−		**−**
**Variable**	**Locoregional Control**							**Distant Metastasis Control**						
	**Univariate (HR[CI])**	***p***		**Multivariate (HR[CI])**		***p***		**Univariate (HR[CI])**			***p***		**Multivariate (HR[CI])**	***p***
**Leukocytosis**	4.46 (2.57–7.70)	< 0.001		1.99 (1.25–3.19)		0.004		2.06 (1.00–4.24)			0.049		2.04 (1.27–3.28)	**0.003**
**Neutrophilia**	3.76 (2.18–6.49)	< 0.001		1.68 (1.03–2.74)		0.038		-			0.069		1.81 (1.11–2.97)	**0.017**
**Monocytosis**	2.87 (1.60–5.16)	< 0.001		-		0.072		5.10 (2.47–10.54)			< 0.001		-	**0.108**
**Anemia**	−	0.620		−		−		−			0.509		−	**-**
**Lymphopenia**	−	0.550		−		−		−			0.688		−	**-**
**Age**	−	0.664		−		−		−			0.932		−	**-**
**PS**	2.06 (1.38−3.10)	< 0.001		1.79 (1.30−2.47)		< 0.001		1.77 (1.00−3.10)			0.048		1.79 (1.29−2.48)	**< 0.001**
**T length > 7cm**	2.46 (1.41−4.31)	0.001		−		0.108		3.27 (1.56−6.83)			0.002		1.77 (1.09−2.88)	**0.021**
**T stage**	2.03 (1.25−3.30)	0.004		1.72 (1.07−2.79)		0.027		−			0.612		−	**-**
**N+ status**	−	0.230		−		−		−			0.620		−	**-**
**IMRT**	−	0.220		−		−		−			0.205		−	**-**
**Tr. duration**	−	0.220		−		−		−			0.098		−	0.915
**RT dose**	−	0.140		−		−		−			0.850		−	

a, Neutrophilia and leukocytosis were not tested in the same model, as neutrophils are subpopulation of leucocytes.

IMRT: Intensity-modulated radiotherapy; Leukocytosis: > 10 G/L; Neutrophilia: > 7 G/L; Monocytosis: > 1 G/L; PS: Performance Status; RT: Radiotherapy; T length: Tumor length;

## DISCUSSION

This study retrospectively evaluated prognostic factors predicting tumor response and survival in patients with locally advanced esophageal SCC treated with exclusive chemoradiation. Poorer outcome was seen in patients with baseline leukocytosis, even after adjusting for other factors.

In this population, which clearly needs therapeutic advances, the challenges are both locoregional and distant control. Attempts to improve locoregional control with higher radiation doses, or locoregional and distant control with concurrent cetuximab or bevacizumab have both failed [10, 11]. Similarly, there were no clear positive improvements when comparing concurrent chemotherapy regimens used. One hypothesis is that these strategies failed because patients were not sufficiently selected, and subgroups of patients might potentially benefit, although these subgroups have still not been identified. Hence, the importance of determining prognostic factors for patient stratification.

Tumor-node-metastasis (TNM) cancer staging was developed to describe the anatomic extent of tumors by Pierre Denoix (Gustave-Roussy Institute) between 1943 and 1952 [12]. Before 1987, tumor length ≤ 5 cm was categorized as T1-status and > 5 cm as T2-status by the 1983 AJCC TNM staging system for esophageal cancer. Its prognostic role was predominant in SCC histologies [12, 13]. Endoscopic tumor length has a significant impact on both the OS and DMC of patients with resected esophagus SCC [14]. Surgical reports displayed that depth invasion of esophageal stratums correlates with tumor length in SCC [15]. Thus, tumor length was replaced with depth of the esophageal wall invasion in the 1987 version of the AJCC TNM staging system and the latest edition (2010) staged ADK and SCC as two different types [10, 12]. A 7 cm cutoff seems logical, knowing that a 5 cm one was recommended to distinguish T1 and T2 disease, and most of our population had locally-advanced disease (93% had T3-4 or N1-2). However, the TNM classification is obviously insufficient for accurately predicting patient outcome.

Chronic inflammation is essential for cancer growth and metastasis, and similarly to in systemic diseases, increased peripheral neutrophil count is associated with worse outcomes in cancer patients, even in the case of a localized tumor [16]. Baseline leukocytosis in Chinese patients with common solid tumors was described in 4–27% of patients, and was associated with a worse outcome [17]. Yet, the incidence of hematological abnormalities in Eastern patients with solid tumors is lower than that of the counterparts in Western countries [17]. Cancer-related inflammation and neutrophils suppress antitumor immunity through regulatory lymphocyte-T cell recruitment, and chemokine activation [4]. Depending on tumor microenvironment and growth phase, neutrophils exert protumoral or antitumoral actions through the production of cytokines (tumor necrosis factor, interleukin-1, and -6) and chemokines, among others [4].

Studies focusing on different cancer types have identified specific prognostic inflammatory markers, including the Glasgow prognostic score (GPS) and neutrophil-to-lymphocyte ratio (NLR). GPS, an inflammation-based prognostic score including serum CRP and albumin levels, has been reported to be valuable in combination with conventional staging techniques to improve the prediction of survival in patients with inoperable gastro-esophageal cancer [18]. In the context of chemoradiation for locally advanced esophageal cancer patients, there is also a correlation between OS and PFS with changes in NLR and PLR among treatment [19]. This is also described in patients undergoing chemoradiation for head and neck SCC [20]. Increased NLR may be associated with poorer survival in patients with esophageal cancer, regardless of treatment regimen (surgery alone, surgery and chemotherapy, chemoradiation), but a meta-analysis displayed that in patients treated with exclusive chemoradiation, NLR wasn't associated with outcome [8, 21]. Our findings evidence that despite a significant correlation between NLR with OS and PFS in univariate analysis, this association seems rather related with neutrophilia than lymphopenia; indeed, lymphopenia was not associated with OS or PFS in our cohort.

Under inflammatory conditions, monocytes may also promote tumor cell spreading through circulation [22]. Classical monocytes promote tumorigenesis and cancer metastasis, while non-classical “patrolling” monocytes (PMo) interact with tumors promoting natural killer cell recruitment, activation, and contribute to cancer immunosurveillance [23]. In patients with esophageal SCC treated with curative surgery, higher preoperative absolute peripheral monocyte count predicts worse OS and DMC, and a low lymphocyte to monocyte ratio predicts poor OS [7, 24]. Our findings also highlight that monocytosis promotes distant relapses. The tumor-monocyte-endothelial axis may also represent a new therapeutic target to reduce cancer metastasis [22]. Enhancement of PMo-mediated immune blockade therapeutically might reduce the already metastatic process below a threshold such that metastasis would not occur [25].

Increased intratumoral and peritumoral neutrophils associated with poor outcome suggests the possible effect of immune misbalance of tumor microenvironment in facilitating esophageal SCC progression, suggesting that immunotherapy should be considered in the future [16]. Contrary to the long held belief that chemotherapy is immunosuppressive, emerging evidence indicates that the anticancer activity of platinum derivatives also has important immunomodulatory effects, highlighting the therapeutic potential of synergistic strategies that combine conventional chemotherapy with immunotherapy [26]. Since inflammation and sex steroids also impact tumorigenesis, a therapeutic approach targeting glucocorticoid receptors and radiation-induced production of tumorigenic factors might be effective in sensitizing SCC to ionizing radiation [9].

The major limitation of our study is its retrospective design. Bi-institutional analysis and strong associations between patient outcome and multiple pretreatment characteristics, including hematological parameters are strengths of this study, as well as the homogenous technique used for radiotherapy. To our knowledge, tumor length, depth of invasion, leukocytosis, neutrophilia, thrombocytosis, and monocytosis have never been investigated as predictive data set in patients undergoing definitive chemoradiation for esophageal SCC. We evidenced that baseline leukocytosis was the strongest independent prognostic factor, ahead of PS, TNM, or tumor length, associated with worse OS, PFS and LRC. We have not explored it in esophageal adenocarcinomas. If future studies confirm this prognostic value of leukocytosis is histologically dependent, it would be of interest to further define the mechanisms of this phenomenon. The next step is to confirm these data in an independent cohort before translating these results to the clinic. However, if validated, these biomarkers might be used for adaptive strategies in order to improve patient outcome.

## MATERIALS AND METHODS

### Patients and tumors

We examined clinical records of consecutive patients treated in 2 French institutions (Gustave Roussy Cancer Campus, Villejuif, France; Paoli-Calmettes Institute, Marseille, France) between April 2003 and August 2015 for histologically confirmed non-operable esophageal SCC, treated with definitive chemoradiation. Data extraction was performed independently in each institution.

Clinical work-up included upper gastrointestinal endoscopy with histologic biopsy and endoscopic ultrasonography; computed tomography (CT) exploring cervical, thoracic, and abdominal regions; positron-emission tomography-CT (PET-CT); and external ultrasonography of the neck with fine-needle aspiration of lymph nodes when cancer was suspected. Disease staging was defined according to the American Joint Committee on Cancer (AJCC) Esophagus and Esophagogastric Junction staging manual, 7th edition [27]. Tumor length was defined by endoscopic ultrasound or CT (whichever was larger). Excluded patients were metastatic, those with a history of prior chemotherapy or radiotherapy, and those treated with a palliative intention by hypofractionated chemoradiation.

### Treatment characteristics

The clinical target volume 1 (CTV 1) was defined from gross tumor volume (GTV T) based on radiologic and endoscopic findings plus a 1.5 cm radial margin and 4–5 cm craniocaudal margin (in case of tumor extension into the stomach, a distal margin of 3 cm) plus the GTV N of involved nodes on PET-CT with a 1 cm margin (CTV N). Reduction boost volume CTV 2 was defined from GTV T plus a 1 cm radial margin and a 2 cm craniocaudal margin. Two planning target volumes (PTVs) were defined, PTV 1 and PTV 2, by adding a uniform 1cm margin to CTV 1 and 2, respectively. Patients received external beam radiotherapy (EBRT) to the prescribed doses 40–41.4 Gy (in 20–23 fractions of 1.8–2 Gy/fraction) to the PTV 1 and 50–66 Gy (at 1.8–2 Gy/fraction) to the PTV 2.

Most patients treated before 2014 underwent a 3D conformal technique using multiple fields with high megavoltage photons generated by a linear accelerator. Those treated after 2014 underwent intensity-modulated radiotherapy (IMRT) with a simultaneous integrated boost (SIB). All patients underwent 3D planning. Organs at risk (OARs) were the spinal cord, the heart, and the lungs. All OARs were delineated to allow the generation of dose-volume histograms and calculation of maximum-tolerated doses. Treatment was performed with a linear accelerator of at least 6 MV with an isocentric technique. The prescription dose was specified at the ICRU50/62 reference point, which was the isocenter for most patients. The daily prescription dose was 1.8–2 Gy at the ICRU reference point. The 95% isodose line encompassed the entire PTV, and the maximum dose to the PTV did not exceed the prescription dose by > 7% (ICRU 50/62 guidelines).

Radiotherapy started on the first day of the first chemotherapy cycle. Patients received concurrent chemotherapy, with four cycles (two concomitant to radiotherapy) of fluorouracil 1000 mg/m^2^ per day for 4 days and cisplatin 75 mg/m^2^ on day 1 (5FU-cisplatin) before 2014, and six cycles (three concomitant to radiotherapy) of oxaliplatin 85 mg/m^2^, leucovorin 200 mg/m^2^, bolus fluorouracil 400 mg/m^2^, and infusion fluorouracil 1600 mg/m^2^ over 46 h (FOLFOX) after 2014, in accordance to the state of the art [3]. Patients unfit for these regimens were treated with concurrent platinum based monotherapy or other regimens.

### Complete blood count analysis

Patients underwent systematic complete blood cell counts weekly during chemoradiation. Pretreatment blood samples taken before any chemotherapy were employed in the current analysis. Leukocytosis and neutrophilia, defining biological inflammation, were defined as blood count over 10 G/L and 7 G/L, respectively. Anemia was defined as hemoglobin count below 12.0 g/dL; thrombocytosis as platelet count over 400,000 /mm3; lymphopenia as lymphocyte count below 1,000 /mm3; and monocytosis as monocyte count over 1,000 /mm3. We excluded from the analysis patients who received neoadjuvant chemotherapy or corticosteroids, presented chronic inflammation, were under treatment for an immune disease, or presented acute or chronic infection (including Human Immunodeficiency Virus). We also excluded patients with a history of neoplasia or medication which may confound the analysis (G-CSF, antibiotics for a recent infection).

### Follow-up and statistical analysis

Follow-up was scheduled at 6 weeks, then every three months during two years, then every 6 months. Systematic CT exploring cervical, thoracic, and abdominal regions, and PET-CT were performed 3 months after CRT completion, then every six months. Differences in patient characteristics regarding baseline neutrophilia were compared by student's *t*-test, chi-2 and by variance analysis. A correlation matrix was constructed using Pearson's correlation coefficient. Factors associated with tumor relapse were examined. Receiver operating characteristic (ROC) curves were constructed for quantitative variables to determine the best cut-off point values discriminating between groups with good or poor prognosis and to define the distinctions between groups with differing prognosis. Survival times were defined as the time between diagnosis and the last follow-up or first event (time of death for OS, recurrence or death for progression free survival [PFS], locoregional recurrence for locoregional control [LRC] and distant metastasis [DM] for distant metastasis control [DMC]) estimated by the Kaplan Meier method. Survival curves were compared using log-rank and the Cox univariate hazards model. Multivariate analyses were performed for variables with *p* value < 0.20 in univariate analysis, according to Cox multivariate proportional hazards model. In multivariate analysis, leukocytosis, neutrophilia and monocytosis were tested in different models, as they are a subpopulation of leucocytes. Statistical analyses were performed using RStudio (2015) (RStudio: Integrated Development for R. RStudio, Inc. Boston, MA; URL http://www.rstudio.com/).

## CONCLUSIONS

These findings suggest the strong prognostic value of baseline leukocytosis and neutrophilia, above previously well-established determinant conditions (PS, TNM, and tumor length) in patients with locally advanced esophageal SCC carcinoma treated with exclusive chemoradiation.

## SUPPLEMENTARY MATERIALS FIGURES AND TABLES



## Abbreviations

5FU: 5-fluorouracil
ADK: Adenocarcinoma
AJCC: American Joint Committee on Cancer
CRP: C - reactive protein
DMC: Distant Metastases Control
CTV: Clinical Target Volume
EBRT: external beam radiotherapy
G-CSF: Granulocyte Colony-Stimulating Factor
GTV: Growth Tumor Volume
Gy: Gray
HIV: Human Immunodeficiency Virus
HPV: Human Papillomavirus
IMRT: Intensity-Modulated Radiotherapy
LRC: Locoregional Control
MLR: Monocyte Lymphocyte Ratio
MRI: MV: Mega Voltage
NLR: Neutrophils to Lymphocytes Ratio
OARs: Organs At Risk
OS: Overall Survival
PET-CT: Positron-emission tomography CT
PFS: Progression Free Survival
PLR: Platelet Lymphocyte Ratio
PMo: Patrolling Monocytes
ROC: Receiver Operating Characteristic
SCC: Squamous Cell Carcinoma
VEGF: Vascular Endothelial Growth Factor
