# Supplementary material for: Leukocytosis and neutrophilia predict outcome in locally advanced esophageal cancer treated with definitive chemoradiation
Source: Oncotarget. 2017 Jan 10;8(7):11579–88. doi: 10.18632/oncotarget.14584 (PMC5355287; doi:10.18632/oncotarget.14584)
Supplement: Supplementary file 1 [file oncotarget-08-11579-s001.pdf]

Leukocytosis and neutrophilia predict outcome in locally advanced esophageal cancer treated with definitive chemoradiation

Supplementary Materials

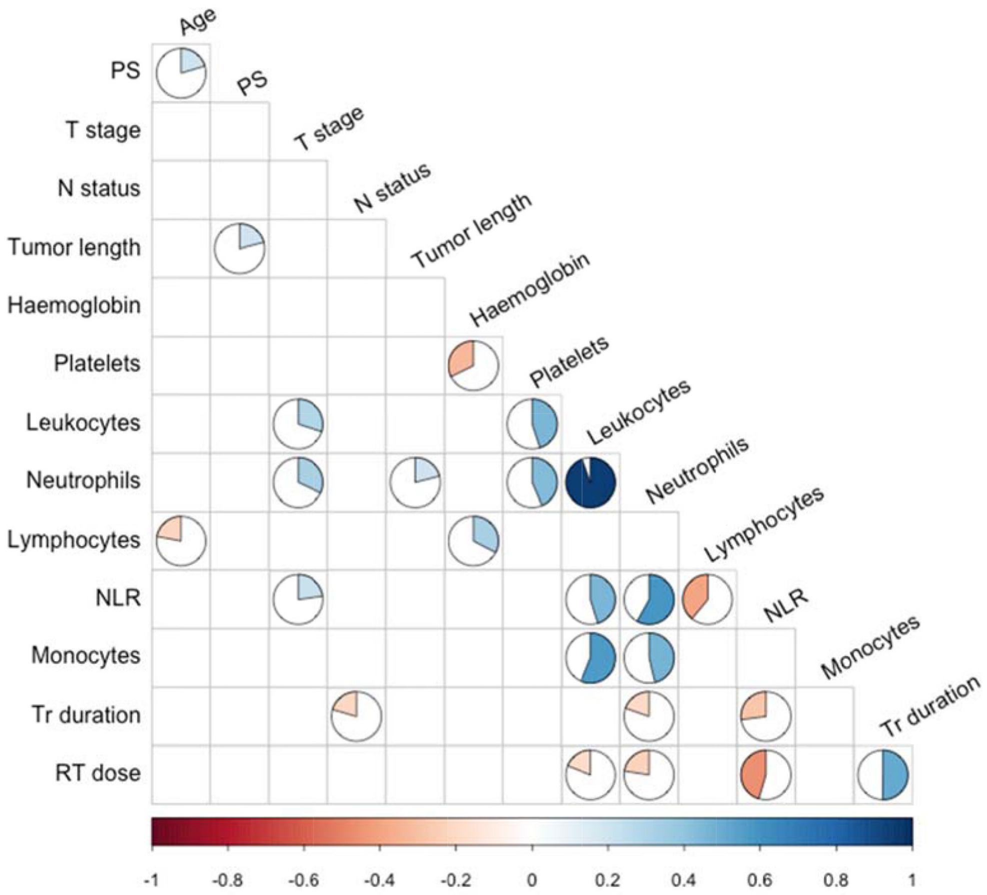

Supplementary Figure 1: Correlation matrix. *all variables considered as quantitative; Pearson's correlation coefficients are represented as pies; unsignificant *p*-values (> 0,05) testing non-correlations are blanked. NLR: Neutrophil to Lymphocyte Ratio; PS: Performance status; RT: radiotherapy; Tr: treatment;

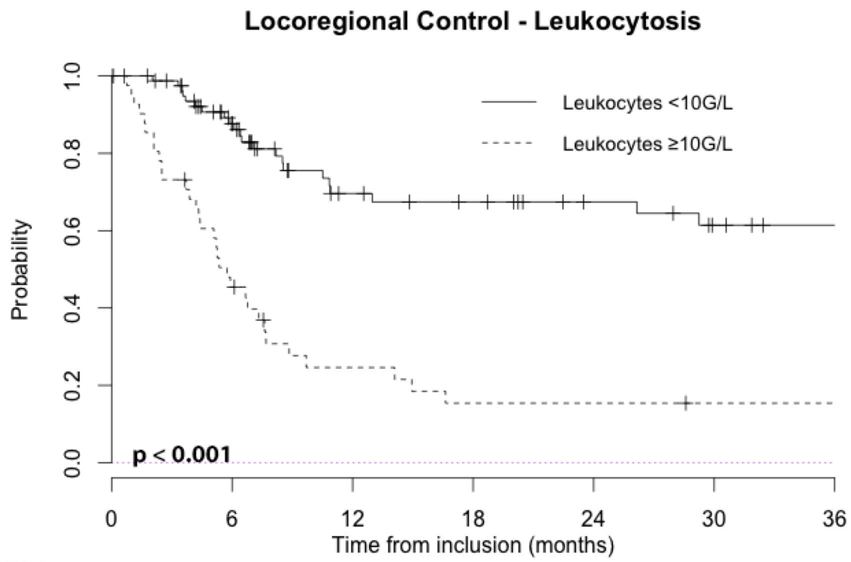

No. At Risk							
Leukocytes <10G/L	84	57	33	29	23	18	15
Leukocytes ≥10G/L	41	17	8	5	5	4	4

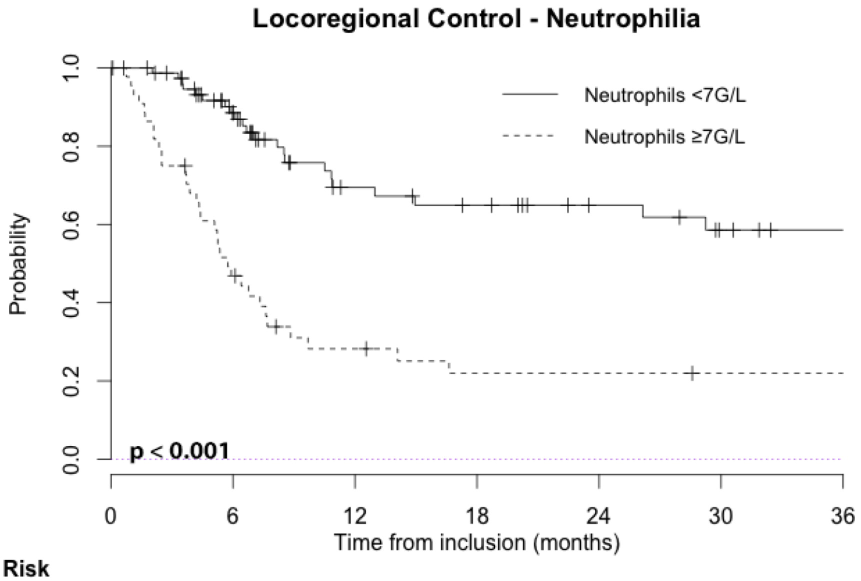

No. At Risk							
Neutrophils <7G/L	81	55	31	27	21	16	13
Neutrophils ≥7G/L	44	19	10	7	7	6	6

Supplementary Figure 2: Estimated locoregional control in patients with leukocytosis or neutrophilia.

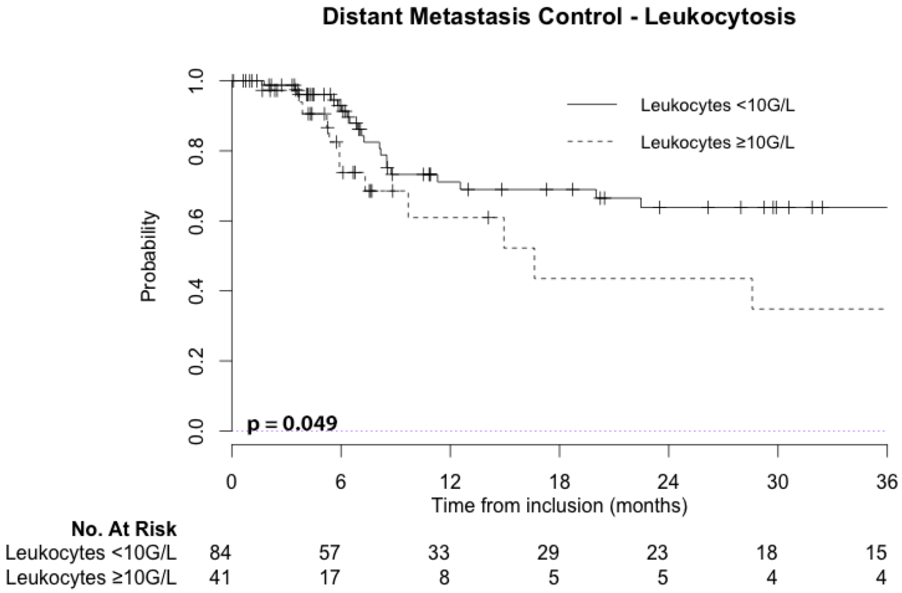

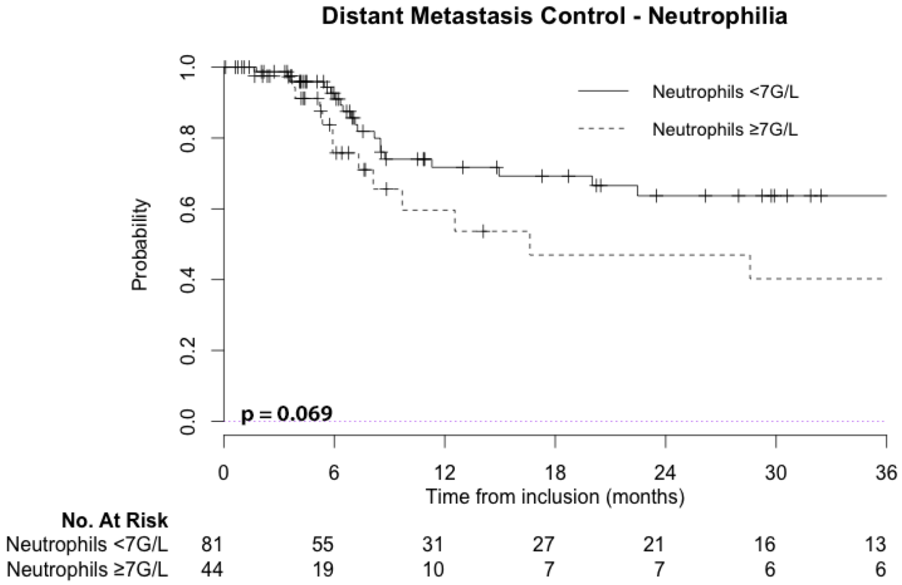

Supplementary Figure 3: Estimated distant metastasis control in patients with leukocytosis or neutrophilia.
